# Probiotic *Pediococcus pentosaceus* restored gossypol-induced intestinal barrier injury by increasing propionate content in Nile tilapia

**DOI:** 10.1186/s40104-024-01011-w

**Published:** 2024-04-07

**Authors:** Feifei Ding, Nannan Zhou, Yuan Luo, Tong Wang, Weijie Li, Fang Qiao, Zhenyu Du, Meiling Zhang

**Affiliations:** Laboratory of Aquaculture Nutrition and Environmental Health (LANEH), School of Life Sciences, East China Normal University, Shanghai, 200241 China

**Keywords:** Gut barrier injury, Gossypol, ISCs proliferation, Nlrc3, Propionate

## Abstract

**Background:**

Intestinal barrier is a dynamic interface between the body and the ingested food components, however, dietary components or xenobiotics could compromise intestinal integrity, causing health risks to the host. Gossypol, a toxic component in cottonseed meal (CSM), caused intestinal injury in fish or other monogastric animals. It has been demonstrated that probiotics administration benefits the intestinal barrier integrity, but the efficacy of probiotics in maintaining intestinal health when the host is exposed to gossypol remains unclear. Here, a strain (YC) affiliated to *Pediococcus pentosaceus* was isolated from the gut of Nile tilapia (*Oreochromis niloticus*) and its potential to repair gossypol-induced intestinal damage was evaluated.

**Results:**

A total of 270 Nile tilapia (2.20 ± 0.02 g) were allotted in 3 groups with 3 tanks each and fed with 3 diets including CON (control diet), GOS (control diet containing 300 mg/kg gossypol) and GP (control diet containing 300 mg/kg gossypol and 10^8^ colony-forming unit (CFU)/g *P. pentosaceus* YC), respectively. After 10 weeks, addition of *P. pentosaceus* YC restored growth retardation and intestinal injury induced by gossypol in Nile tilapia. Transcriptome analysis and siRNA interference experiments demonstrated that NOD-like receptors (NLR) family caspase recruitment domain (CARD) domain containing 3 (Nlrc3) inhibition might promote intestinal stem cell (ISC) proliferation, as well as maintaining gut barrier integrity. 16S rRNA sequencing and gas chromatography-mass spectrometry (GC-MS) revealed that addition of *P. pentosaceus* YC altered the composition of gut microbiota and increased the content of propionate in fish gut. In vitro studies on propionate’s function demonstrated that it suppressed *nlrc3* expression and promoted wound healing in Caco-2 cell model.

**Conclusions:**

The present study reveals that *P. pentosaceus* YC has the capacity to ameliorate intestinal barrier injury by modulating gut microbiota composition and elevating propionate level. This finding offers a promising strategy for the feed industry to incorporate cottonseed meal into fish feed formulations.

**Graphical Abstract:**

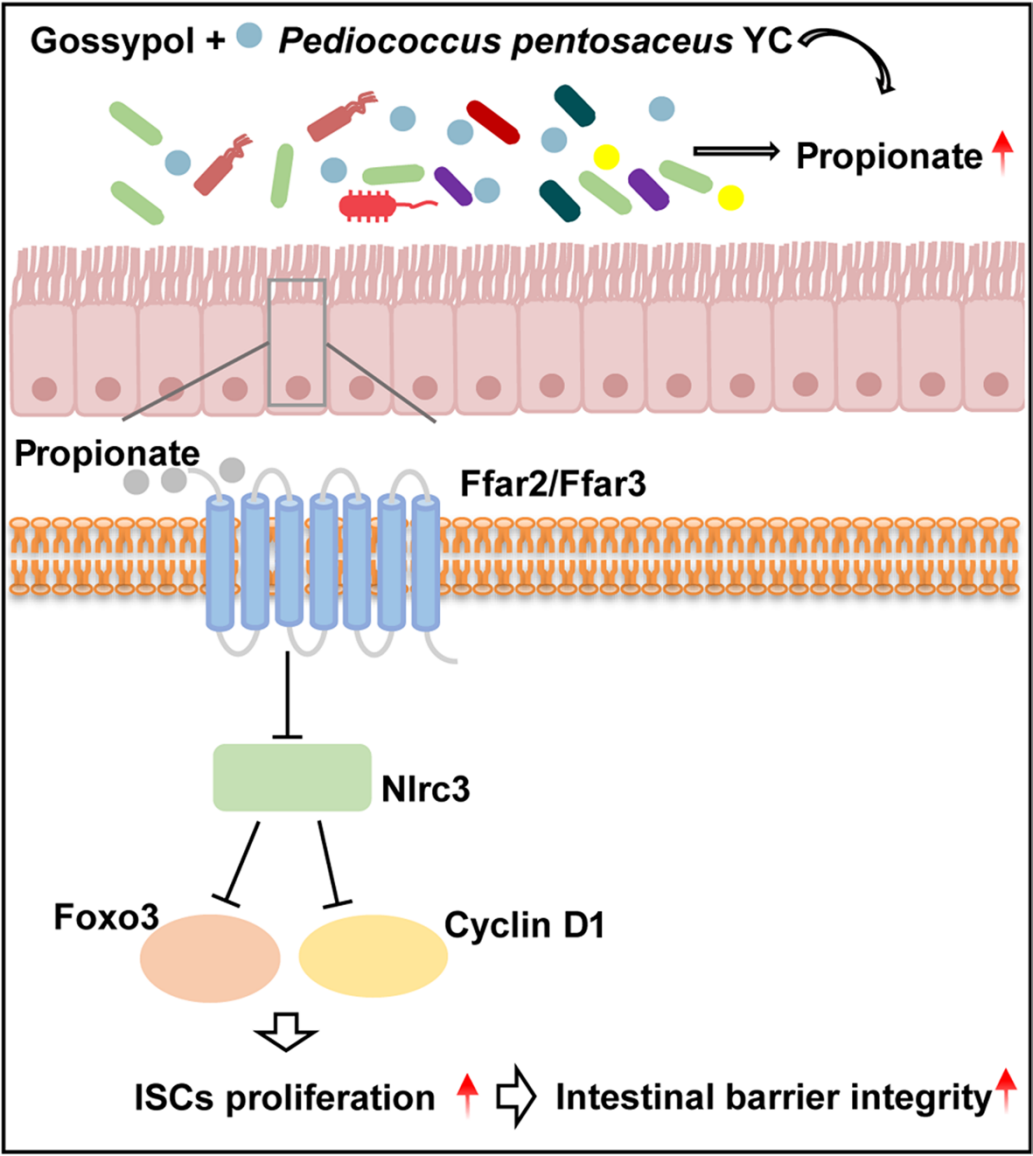

**Supplementary Information:**

The online version contains supplementary material available at 10.1186/s40104-024-01011-w.

## Background

The gastrointestinal tract acts as an important barrier to separate the body from the food components, antigens, intestinal microbiota and other possible toxins. It enables the absorption of nutrients and inhibits the invasion of the potentially harmful compounds or pathogens from the intestinal lumen [1–3]. It has been revealed that some dietary components or xenobiotics impair the intestinal barrier and disrupt the intestinal microbiota composition which in turn exacerbates this process [4, 5]. It has been established that the damage to the intestinal barrier is frequently accompanied by the activation of pro-inflammatory immune cells, then exacerbating the intestinal inflammation [6, 7]. And the impaired gut barrier is unable to prevent lipopolysaccharide (LPS) and other bacterial toxins from entering the circulatory system, thereby promoting hepatitis, hepatoma and meningitis [8–10]. Thus, the dysfunction or impairment of intestinal barrier will trigger intestinal disease or other multi-organ dysfunction syndromes [11].

The animal industry has confronted the challenge of escalating feed costs and the scarcity of protein resources [12, 13]. The cost of feed is largely attributed to the protein sources, so finding cheaper protein alternatives would be advantageous for both the industry and farmers. Cottonseed is one of the major products of cotton, with a global annual production of up to 26 million tons. Cottonseed meal (CSM) is a protein-rich product that can be found in large quantities and is typically more cost-effective compared to other protein sources such as fish meal (FM) and soybean meal (SBM) [14–16]. However, deepening researches have revealed that CSM especially the gossypol residue contained in CSM could cause severe intestinal inflammation and gut barrier injury in diverse monogastric animals such as fishes and livestock [13, 17–24]. Try to decrease the remaining gossypol in CSM is one of the important strategy to eliminate the deleterious effects [25, 26], but it depends on the dephenolization process which may cause extra cost. Our previous research indicated that intestinal microbiota mediated the gossypol-induced gut barrier injury in Nile tilapia (*Oreochromis niloticus*) [21], thus we hypothesized that regulating intestinal microbiota may have the potential to repair gossypol-induced gut barrier injury.

Probiotics are initially employed as an economical and green substitute for antibiotics in feed additives [27, 28], and the Chinese Ministry of Agriculture and Rural Affairs has granted approval for the supplementation of animal feed with *Pediococcus pentosaceus*. Previous research elucidated that administration of *P. pentosaceus* CECT 8330 could increase the abundance of *Bifidobacterium* and *Lactobacillus* to strengthen mucosal integrity in DSS-induced colitis mice [29]. Addition of *P. pentosaceus* ZJUAF-4 restored gut microbiota composition against diquat-induced intestinal injury in mice [30]. *P. pentosaceus* PR-1 increased the abundance of Fusobacteria, *Cetobacterium* and *Plesiomonas* and reinforced gut barrier integrity in high-fat-diet-fed zebrafish (*Danio rerio*) [31]. The intestinal epithelium regenerates every 3–5 d to replenish the aged and damaged cells in the villi and ensure intestinal barrier integrity. Intestinal stem cells (ISCs) express the cell-surface markers, leucine-rich repeat-containing G-protein coupled receptor 5 (Lgr5) and olfactomedin 4 (Olfm4), and differentiate perpetually into intestinal epithelial cells, which governed the renewal of intestinal epithelium [32–35]. Addition of probiotics have been demonstrated to reinforce gut barrier integrity by promoting ISCs proliferation in mice [34, 36], but the exact mechanism by which *P. pentosaceus* repairs the injured intestinal barrier remains unclear.

Nile tilapia is the third most farming teleost fish worldwide [37] and CSM is regarded as the potential protein source for fish in near future. A bacterial strain (YC) affiliated to *P. pentosaceus* was isolated from the gut of Nile tilapia. *P. pentosaceus* YC was used to treat Nile tilapia exposed to gossypol and the restoration effect of *P. pentosaceus* on intestinal permeability was evaluated.

## Methods

### Bacteria strain and culture

The preserved strain *P. pentosaceus* YC, isolated from the gut of Nile tilapia [38], was recovered and spread on the Man Rogosa Sharpe (MRS)-agar plate (Basebio, Hangzhou, China). The bacterial suspension was identified by sequencing the full length of 16S rRNA gene (Personalbio, Shanghai, China). The single colony of *P. pentosaceus* YC was cultured overnight in MRS medium at 28 °C at 100 r/min for 18 h. Following centrifugation at 2,000 × *g* for 15 min, the bacterial precipitation was resuspended in sterile 1 × phosphate buffered saline (PBS) and mixed with the diet powder to form the pellets. The bacterial quantity was detected by serial dilution and counting on MRS agar plates.

### Animal experiments

Juvenile Nile tilapia were purchased from Guangzhou Tianfa Fry Development Co., Ltd. (Guangzhou, China). All fish were raised in the environment with water temperature of 28 °C, a dissolved oxygen higher than 6.0 mg/L, and light/dark cycle of 12 h/12 h. Fish were acclimated and fed with a commercial diet purchased from Tongwei Co., Ltd. (Chengdu, China) for 2 weeks. Two hundred and seventy Nile tilapia (2.20 ± 0.02 g) were randomly divided into nine 200-L tanks (30 fish/tank, 3 tanks/diet), fed with three diets including the CON (control diet), GOS (control diet containing 300 mg/kg gossypol) and GP (control diet containing 300 mg/kg gossypol and 10^8^ colony-forming unit (CFU)/g *P. pentosaceus* YC). During the experiment, fish were fed with gossypol for 8 weeks in GOS group, and fish were fed with *P. pentosaceus* YC daily for 2 weeks prior to the gossypol addition in GP group. All fish were fed twice (08:30 and 18:30) daily and fed at 4% of their average body weight within a day. The formulation of the diets was listed in Additional file 1: Table S1.

### Sample collection

After the feeding trail, Nile tilapia were fasted for 16 h before sampling. Twelve fish were randomly selected from each group (4/tank) and anesthetized with 20 mg/L tricaine methanesulfonate (MS-222, E10521, Sigma-Aldrich, St. Louis MO, USA). The whole intestinal contents were collected for 16S rRNA sequencing, short chain fatty acids (SCFAs) and lactate quantification. Blood samples were drawn from the caudal part of fish and centrifuged at 3,000 r/min for 10 min at 4 °C to obtain serum for LPS concentrations detection. Proximal intestine (PI) and distal intestine (DI) were separately stored at −80 °C for gene expression quantification and transcriptome sequencing. The final body weight, liver, intraperitoneal fat and final body length of each fish were measured and the growth indicators and organ indices were calculated following the under formula:

$$\rm{Weight}\ \rm{gain}\ \left(\rm{WG},\rm{g}\right)=\rm{final}\ \rm{body}\ \rm{weight}\ \left(\rm{FBW},\rm{g}\right)-\rm{initial}\ \rm{body}\ \rm{weight}\ \left(\rm{IBW},\rm{g}\right)$$

$$\text{Specific growth rate}\,\left(\rm{SGR},\%/\mathrm{d}\right)=100\times \left[\left(\ln\ \rm{FBW}-\ln\ \rm{IBW}\right)/ \text{time (d)}\right]$$

$$\rm{Condition}\ \rm{factor}\ \left(\rm{CF},\rm{g}/\rm{cm}3\right)=100\times \left[\rm{body}\ \rm{weight}\ \left(\rm{g}\right)/ \rm{body}\ {\rm{length}}^3\ \left(\rm{cm}\right)\right]$$

$$\rm{Hepatosomatic}\ \rm{index}\ \left(\rm{HSI},\%\right)=100\times \left[\rm{liver}\ \rm{weight}\ \left(\rm{g}\right)/ \rm{body}\ \rm{weight}\ \left(\rm{g}\right)\right]$$

$$\rm{Mesenteric}\ \rm{fat}\ \rm{index}\ \left(\rm{MFI},\%\right)=100\times \left[\rm{mesenteric}\ \rm{fat}\ \rm{weight}\ \left(\rm{g}\right)/ \rm{body}\ \rm{weight}\ \left(\rm{g}\right)\right]$$

### Intestinal permeability assay

Intestinal permeability was detected by an Ussing chamber as previously described [39]. Six proximal intestine segments larger than 0.01 cm^2^ from each group were directly rinsed with the buffer (NaCl, 140 mmol/L; NaHCO_3_, 10 mmol/L; KCl, 4 mmol/L; NaH_2_PO_4_, 2 mmol/L; MgSO_4_, 1 mmol/L; CaCl_2_, 1 mmol/L; glucose, 5.5 mmol/L; pH 7.8) and fixed on a clamp. After an equilibration for 20 min, transepithelial electrical resistance (TEER) was automatically monitored in a 10-min-circuit current.

### Histological analysis

The 4% paraformaldehyde-fixed proximal intestine samples were embedded in paraffin, and then cut into 5 μm slice for the hematoxylin and eosin (H&E) staining. A light microscope (Nikon Ds-Ri2, Nikon Corporation, Tokyo, Japan) was used to image the intestinal villus height, villus thickness and basal layer thickness from at least 24 segments. Quantification and statistical analysis were conducted according to the previous article by using imaging software (Nis-Elements F package version 4.60) [40].

### Biochemical analysis

The LPS concentration in the serum was quantified by using fish LPS enzyme-linked immunosorbent assays (ELISA) kits (HB794-QT, Shanghai Hengyuan Biotechnology Co., Ltd., China). The levels of lactate in intestinal contents were measured using the commercial kit (A019-2-1, Nanjing Jiancheng Bioengineering Institute, Nanjing, China) according to the manufacturer’s instruction.

### Short chain fatty acids quantification

Total SCFAs in intestinal contents were determined using gas chromatography-mass spectrometry (GC-MS). Briefly, 0.02 g intestinal contents mixed with 200 μL 1 × PBS, acidified with 50 μL of 50% sulfuric acid and crushed by homogenizer (DLAB Scientific Co., Ltd., Beijing, China). 250 μL of diethyl ether was used for SCFAs extraction. All operations were performed on ice. The Gas Chromatography Nexis GC-2010 (Shimadzu, Kyoto, Japan) was utilized to measure SCFAs levels according to the following program: temperature increased from 60 °C to 100 °C at a rate of 5 °C/min, for 2 min; increased to 180 °C at 5 °C/min for 2 min. The external standard method was employed in order to calculate the concentration of SCFAs (acetate, propionate and butyrate) (71251, 94425 and 19215, Sigma-Aldrich, St. Louis MO, USA).

### Quantitative PCR (qPCR) analysis

Total RNA from tissues or cells was extracted using Tripure Reagent (RN0102, Aidlab, Beijing, China). The quality and quantity of RNA were determined by agarose gel electrophoresis and NanoDrop 2000 spectrophotometry (Thermo Scientific, Waltham, USA). Then the complementary DNA (cDNA) was synthesized using a FastQuant reverse-transcribed kit with gDNase (R323-01, Nanjing Vazyme Biotech Co., Ltd., Nanjing, China). Quantitative real-time PCR was performed on CFX96 Real-Time PCR system (Bio-Rad, Richmond, USA) using 2 × SYBR Master Mix (Q711-02, Nanjing Vazyme Biotech Co., Ltd., Nanjing, China) containing 10 μmol/L gene specific primer. Gene expression levels were calculated using 2^-ΔΔCT^ method and normalized to the housekeeping genes elongation factor 1 alpha (*ef1α*) and *β-actin*. The PCR primers were designed using NCBI Primer BLAST based on the NCBI database and synthesized by Shanghai Personal Biotechnology Co., Ltd. Primer sequences were summarized in Table S2.

### RNAseq analysis

Total RNA from the distal intestine was extracted, qualified, paired and the purified RNA was used to construct the library using Illumina Truseq^TM^ RNA sample prep kits. Paired-end sequencing was performed on the Illumina Novaseq 6000 sequencing platform (Majorbio, Shanghai, China). The gene expression levels were quantitatively analyzed by RSEM software with TPM as the quantitative index. The differentially expressed genes (DEGs) were analyzed by DESeq2. Genes of *P*-value < 0.05 were considered as DEGs. Genes with detection values higher than 0.1 were used for Kyoto Encyclopedia of Genes and Genomes (KEGG) enrichment analysis and volcano plot analysis. The raw data was available in the NCBI with the BioProject accession number PRJNA987992.

### Gut microbiota analysis

Genomic DNA extracted from the intestinal contents was performed by the Illumina NovaSeq 6000 System (Personalbio, Shanghai, China). Microbial composition was analyzed by targeting the V3–V4 region of 16S rRNA gene using primers 338 F (5′-ACTCCTACGGGAGGCAGCA-3′) and 806 R (5′-GGACTACHVGGGTWTCTAAT-3′). QIIME2 software was used to analyze sequencing reads. Alpha diversity indexes (Chao1, Shannon and Faith_pd) were significantly different among groups as assessed using the Kruskal-Wallis test. Beta diversity was conducted by the principal coordinate analysis (PCoA) based on Bray-Curtis distance. Linear discriminant analysis effect size (LEfSe) analysis was used to characterize taxonomic units with significant differences based on Wilcoxon test. Circos analysis was performed by the Genes cloud tools of Personalbio. The raw data of intestinal microbiota were available in the NCBI with the BioProject accession number PRJNA987999.

### In vivo NOD-like receptors (NLR) family caspase recruitment domain (CARD) domain containing 3 (*nlrc3*) siRNA in the intestine of Nile tilapia

Three siRNA fragments of *nlrc3* (Gene ID: 100694916) were designed to target different encoding regions and the scrambled siRNA (Table S3) was administered to juvenile Nile tilapia (1.11 ± 0.02 g) as previously reported [41]. Briefly, 10 μL of 1 × PBS, siRNA scramble (50 μmol/L) and si*nlrc3* (50 μmol/L) were delivered into the etherized juvenile fish via oral gavage using 10 μL micro pipette tips. The siRNAs were given orally to the same fish every 2 d. Five fish in each group were dissected for mRNA expression analysis at 1 d and 7 d post-siRNA treatment.

### Western blot

Intestinal tissues were homogenized on ice by using ice-cold radioimmunoprecipitation assay (RIPA) lysis buffer (P0013B, Beyotime Biotechnology, Shanghai, China) containing 1 mmol/L phenylmethylsulfonyl fluoride (PMSF) (ST506, Beyotime Biotechnology, Shanghai, China) for 30 min. The extracted protein was mixed with 5 × sodium dodecyl sulfate (SDS) loading buffer and boiled at 95 °C for 10 min. The 100 μg protein was subjected to 10% sodium dodecyl sulfate polyacrylamide gel electrophoresis (SDS-PAGE). The protein concentration was determined using Bicinchoninic acid (BCA) assay Protein Assay kit (P0012, Beyotime Biotechnology, Shanghai, China). The antibodies were as follows: anti-Nlrc3 (DF13411, Affinity Biosciences, Jiangsu, China), anti-α-Tubulin (AF4651, Affinity Biosciences, Jiangsu, China), and the secondary antibody IRDye^®^ 600CW and IRDye^®^ 800CW (Li-Cor Biotechnology, Nebraska, USA). Visualization was carried out using Odyssey Clx (Li-Cor Biotechnology, Nebraska, USA) and the densitometric quantification was performed using Image Studio Lite Ver 5.2 software.

### Caco-2 cell line culture and treatments

Briefly, Caco-2 cells were obtained from ATCC, maintained in medium (RPMI 1640, 20% FBS, 100 U/mL penicillin, 0.1 mg/mL streptomycin) in a humidified incubator (37 °C and 5% CO_2_) and passaged every 2–3 d at 80% confluency. For gene expression detection, cells were incubated in 12-well plates with gossypol (20 μmol/L) and sodium propionate (SP) (1 mmol/L and 5 mmol/L) for 24 h. For wound-healing assay, cells were seeded in 12-well plates until reaching 90%–100% confluency. After serum starving, 10 μL pipette tip was used to make a scratch in cell monolayer. Cells were treated with gossypol (20 μmol/L) and SP (1 mmol/L and 5 mmol/L) for 72 h. An inverted light microscope (Nikon Ds-Ri2, Nikon Corporation, Tokyo, Japan) was used to image and the imaging software (Nis-Elements F package version 4.60) was used to measure the wound closure. The wound closure was calculated according to the formula: [(original area of wound − final area of wound)/original area of wound]/2.

### Statistical analysis

Data were presented as mean ± standard error of mean (SEM). Shapiro−Wilk test and Levene’s test were used to test the normality and the homogeneity of variances for all data. One-way analysis of variance (ANOVA) with Tukey’s adjustment was used to compare the differences among groups and unpaired Student’s* t*-test was conducted for the difference analyses between two groups in GraphPad Prism 8. A value of *P* < 0.05 was deemed statistically significant.

## Results

### The effects of *P. pentosaceus* YC on the growth performance

After the feeding trial, the growth parameters were recorded to analyze the effects of *P. pentosaceus* YC on the growth performance of Nile tilapia. The WG, SGR and FBL were significantly decreased in GOS group when compared to the CON group, and they were significantly increased in GP group compared with the GOS group (*P* < 0.05, Table 1). The CF, HSI and MFI had no significant difference among three groups (*P* > 0.05, Table 1). Collectively, addition of *P. pentosaceus* YC enhanced the growth performance of Nile tilapia.

**Table 1:** Effects of three diets on growth performance in Nile tilapia

Items	CON	GOS	GP
Initial body weight, g	2.20 ± 0.02	2.20 ± 0.02	2.20 ± 0.02
Weight gain, g	27.98 ± 0.43^a^	24.78 ± 0.37^b^	27.42 ± 0.05^a^
Specific growth rate, %/d	3.19 ± 0.01^a^	2.99 ± 0.03^b^	3.14 ± 0.03^a^
Final body length, cm	11.85 ± 0.21^a^	10.52 ± 0.22^b^	11.79 ± 0.13^a^
Condition factor, g/cm^3^	1.60 ± 0.04	1.53 ± 0.03	1.54 ± 0.02
Hepatosomatic index, %	2.32 ± 0.09	1.99 ± 0.12	2.01 ± 0.12
Mesenteric fat index, %	0.38 ± 0.05	0.29 ± 0.04	0.31 ± 0.04

Data are represented as mean ± SEM and analyzed by ANOVA with Tukey test. ^a,b^Different lowercase letters indicated significant differences (*P* < 0.05)

### Addition of *P. pentosaceus* YC repaired gossypol-induced intestinal barrier injury

**Fig. 1:**
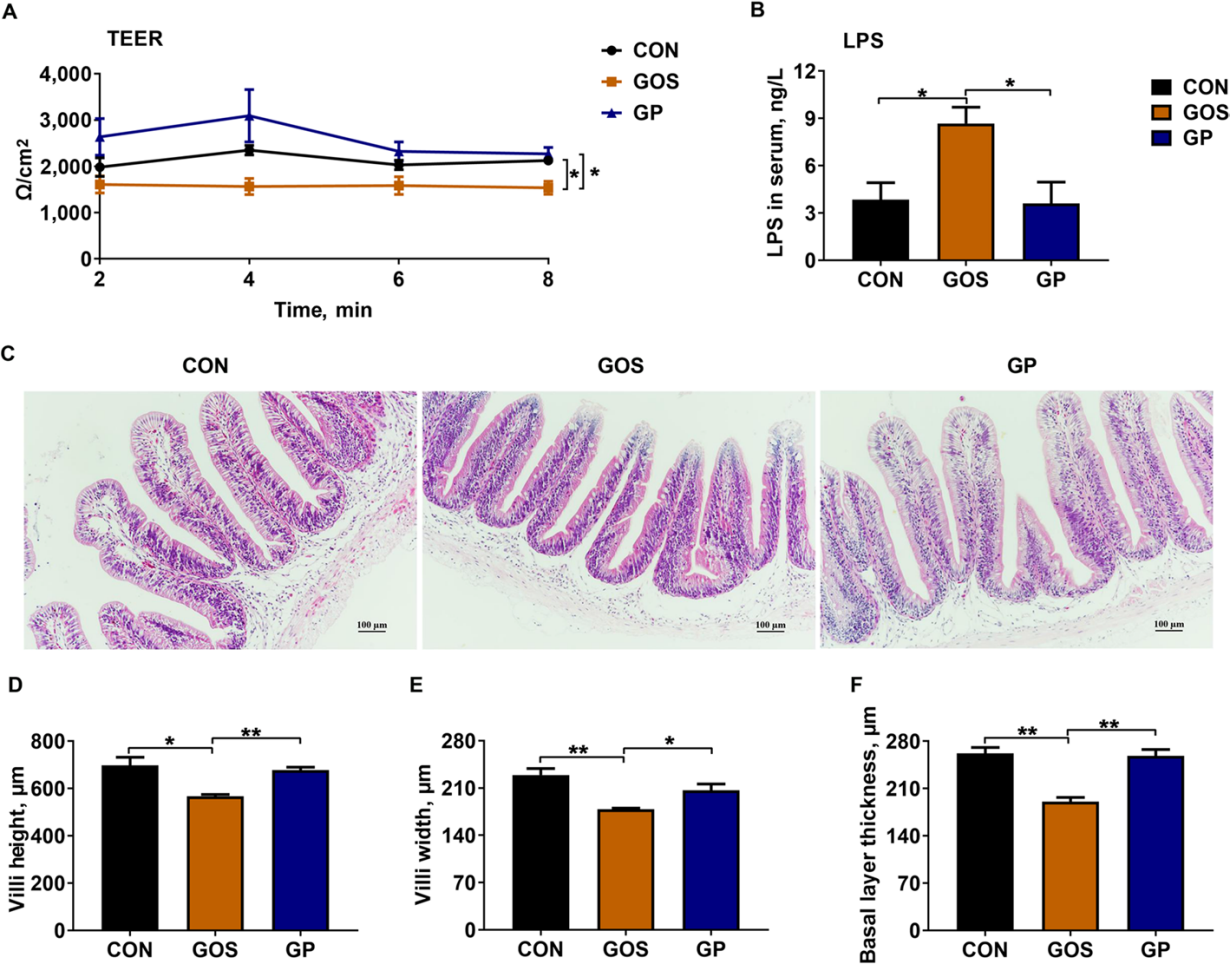
Addition of *P. pentosaceus* YC repaired gossypol-induced intestinal barrier injury. **A** The TEER of intestine; **B** The LPS concentrations in serum (*n* = 6 individuals); **C** H&E straining of intestine; **D** Villi height; **E** Villi width; **F** Basal layer thickness (*n* = 24 individuals). Data are represented as mean ± SEM. Asterisk refers to the significant difference (ANOVA with Tukey’s test; ^*^P < 0.05, ^**^P < 0.01). CON, control diet; GOS, gossypol diet; GP, gossypol diet supplemented with *P. pentosaceus* YC; TEER, transepithelial electrical resistance; LPS, lipopolysaccharide

We further detected the intestinal barrier integrity of Nile tilapia. The TEER was significantly reduced in GOS group and remarkably increased in GP group (*P* < 0.05, Fig. 1A). Consistently, serum LPS concentration was significantly increased in GOS group and decreased after the addition of *P. pentosaceus* YC (*P* < 0.05, Fig. 1B), suggesting an increase of intestinal barrier permeability [42, 43]. Dietary gossypol caused shedding of intestinal epithelial cells at the top of villi, while addition of *P. pentosaceus* YC ameliorated the epithelium damage (Fig. 1C). Gossypol significantly decreased villi height, villi width and basal layer thickness (*P* < 0.05), while addition of *P. pentosaceus* YC resulted in a remarkable improvement of these indicators (*P* < 0.05, Fig. 1D–F). These results indicated that addition of *P. pentosaceus* YC repaired gossypol-induced gut barrier injury in Nile tilapia.

### Addition of *P. pentosaceus* YC increased the gene expression of tight junction proteins (tjps)

**Fig. 2:**
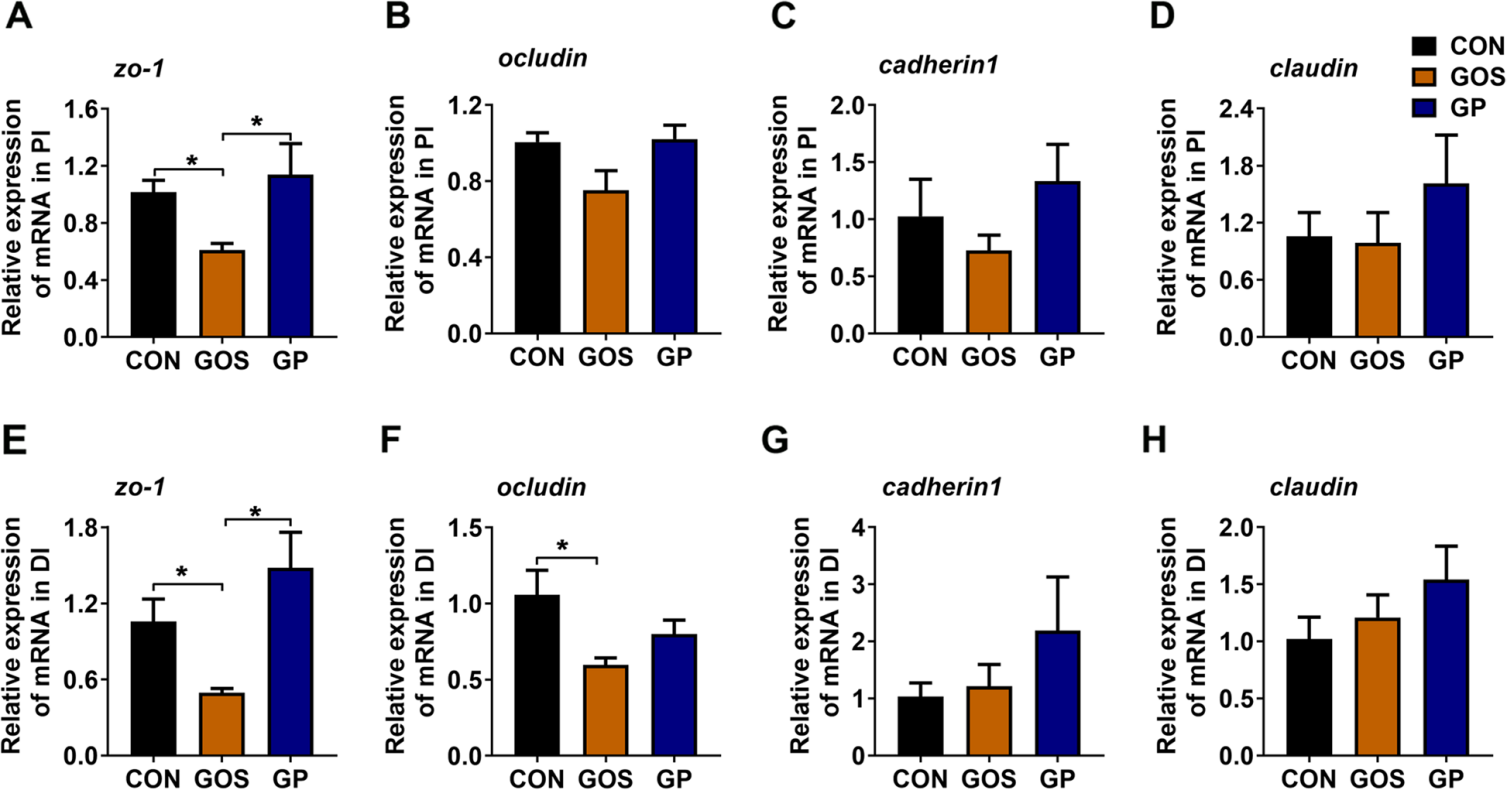
Addition of *P. pentosaceus* YC promoted tjps expression. **A–D** The relative gene expression of *zo-1*, *ocludin*, *cadherin1* and *claudin* in PI; **E–H** The relative gene expression of *zo-1*, *ocludin*, *cadherin1* and *claudin* in DI (*n* = 6). Asterisk refers to the significant difference (ANOVA with Tukey’s test; ^*^P < 0.05). CON, control diet; GOS, gossypol diet; GP, gossypol diet supplemented with *P. pentosaceus* YC; *zo-1*, zona occludens 1; PI, proximal intestine; DI, distal intestine

Epithelial cell tjps such as zona occludens 1 (*zo-1*), *ocludin, cadherin1* and *claudin* suppported the integrity of the gut barrier, and the decrease of their expression can lead to a higher permeability of the intestine [44, 45]. In this study, we found that the expression level of *zo-1* was significantly reduced in PI and DI when fish fed with gossypol (*P* < 0.05, Fig. 2A and E), but it was markedly elevated after addition of *P. pentosaceus* YC (*P* < 0.05, Fig. 2 A and E). Moreover, the gene expression of *ocludin* was significantly decreased in DI when fish fed with gossypol (*P* < 0.05, Fig. 2F). There was no significant difference in the expression of *ocludin* in PI (*P* > 0.05, Fig. 2B), *cadherin1* (*P* > 0.05, Fig. 2C and G) and *claudin* (*P* > 0.05, Fig. 2D and H) in PI and DI among three groups.

### Addition of *P. pentosaceus* YC inhibited Nlrc3 and promoted the gene expression of ISC markers

**Fig. 3:**
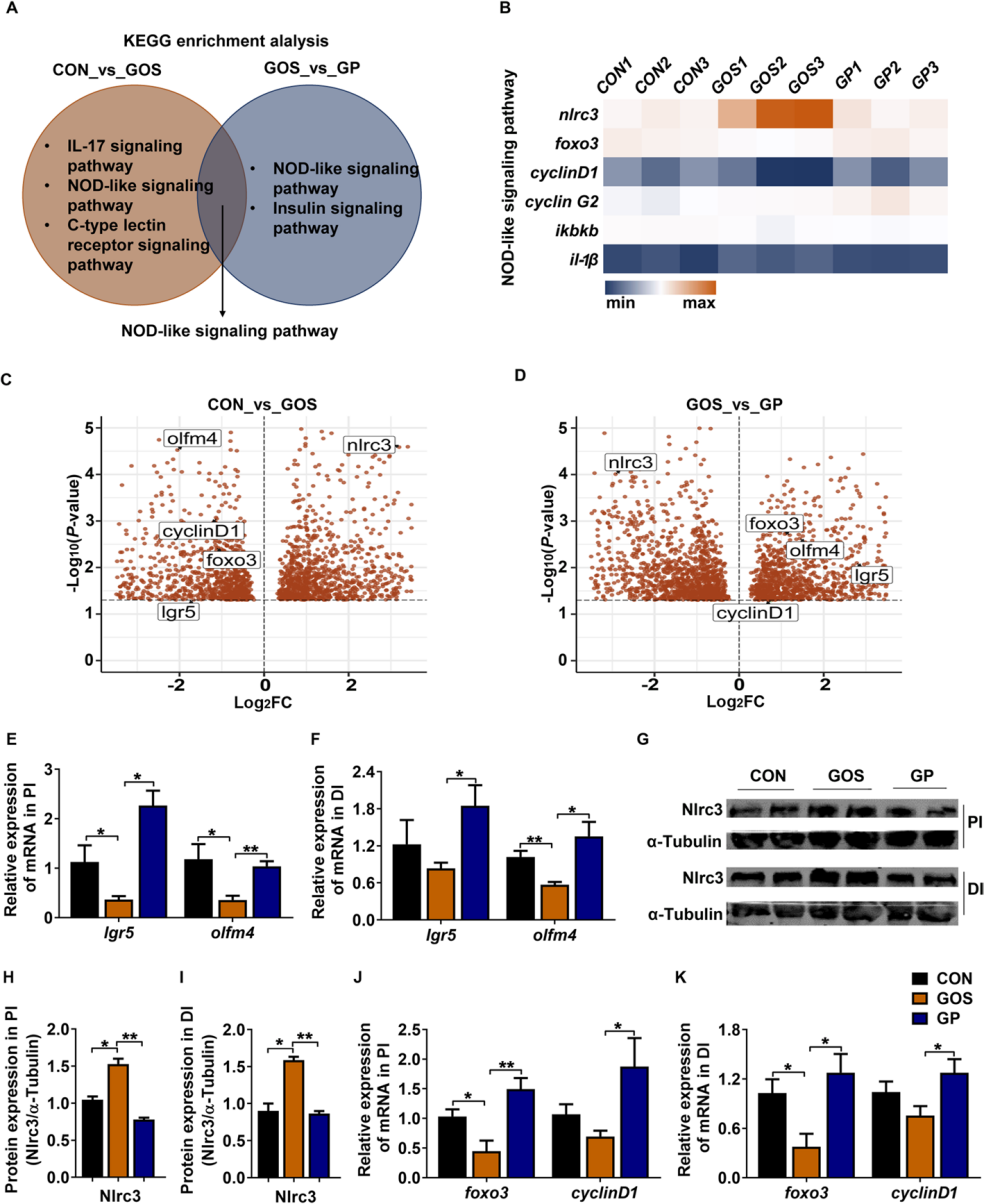
Addition of *P. pentosaceus* YC inhibited Nlrc3 and promoted the gene expression of ISC markers. **A** KEGG enrichment analysis; **B** Heatmap of genes of NOD-like signaling pathway; **C** and **D** Volcano plot of DEGs in the compared groups (*n* = 3). **E** and **F** The relative gene expression of ISC markers (*lgr5* and *olfm4*) in PI and DI; **G** The protein expression of Nlrc3 in PI and DI; **H** and **I** The quantification of the protein expression of Nlrc3 in PI and DI; **J** and **K** The relative gene expression of *foxo3* and *cyclinD1* in PI and DI (*n* = 6). Data are represented as mean ± SEM. Asterisk refers to the significant difference (ANOVA with Tukey’s test; ^*^P < 0.05, ^**^P < 0.01). CON, control diet; GOS, gossypol diet; GP, gossypol diet supplemented with *P. pentosaceus* YC; FC, fold change; *lgr5*, leucine-rich repeat-containing G protein-coupled receptor 5; *olfm4*, olfactomedin 4; *nlrc3*, NLR family CARD domain containing 3; *foxo3*, forkhead box O3; *iκbκb*, inhibitor of nuclear factor kappa B kinase; *il-1β*, interleukin-1β; PI, proximal intestine; DI, distal intestine

The RNAseq analysis was further used to explore the possible mechanism by which *P. pentosaceus* YC repaired gossypol-damaged intestinal barrier. The KEGG enrichment analysis enriched a shared NOD-like signaling pathway (Fig. 3A) which included 4 cell proliferation related genes (*nlrc3*, forkhead box O3 (*foxo3*), *cyclinD1, cyclin G2*) and inflammatory related genes (inhibitor of nuclear factor kappa B kinase (*iκbκb*), interleukin-1β (*il-1β*)) (Fig. 3B)*.* The expression of *nlrc3* was significantly increased in GOS group and dramatically decreased in GP group (*P* < 0.05, Fig. 3B–D). The expression of *foxo3* and *cyclinD1* was significantly decreased in GOS group and remakedly increased in GP group (*P* < 0.05, Fig. 3B–D). We observed that dietary gossypol significantly decreased the expression of ISC marker genes *lgr5* and *olfm4* (*P* < 0.05, Fig. 3C), but addition of *P. pentosaceus* YC remarkly increased their expressions (*P* < 0.05, Fig. 3D), indicating *P. pentosaceus* YC administration might promote ISCs proliferation. The expression of ISC marker genes (*lgr5* and *olfm4*) (Fig. 3E and F), the NOD-like signaling pathway related genes (*nlrc3, foxo3* and *cylinD1*) (Fig. 3 G–K) in PI and DI were confirmed by qPCR and Western blot, and the results were consistent with the transcriptome analysis. Together, these data indicated that addition of *P. pentosaceus* YC inhibited Nlrc3 expression and promoted the gene expression of ISC markers.

### Nlrc3 was the key factor to regulate genes related to ISC markers

**Fig. 4:**
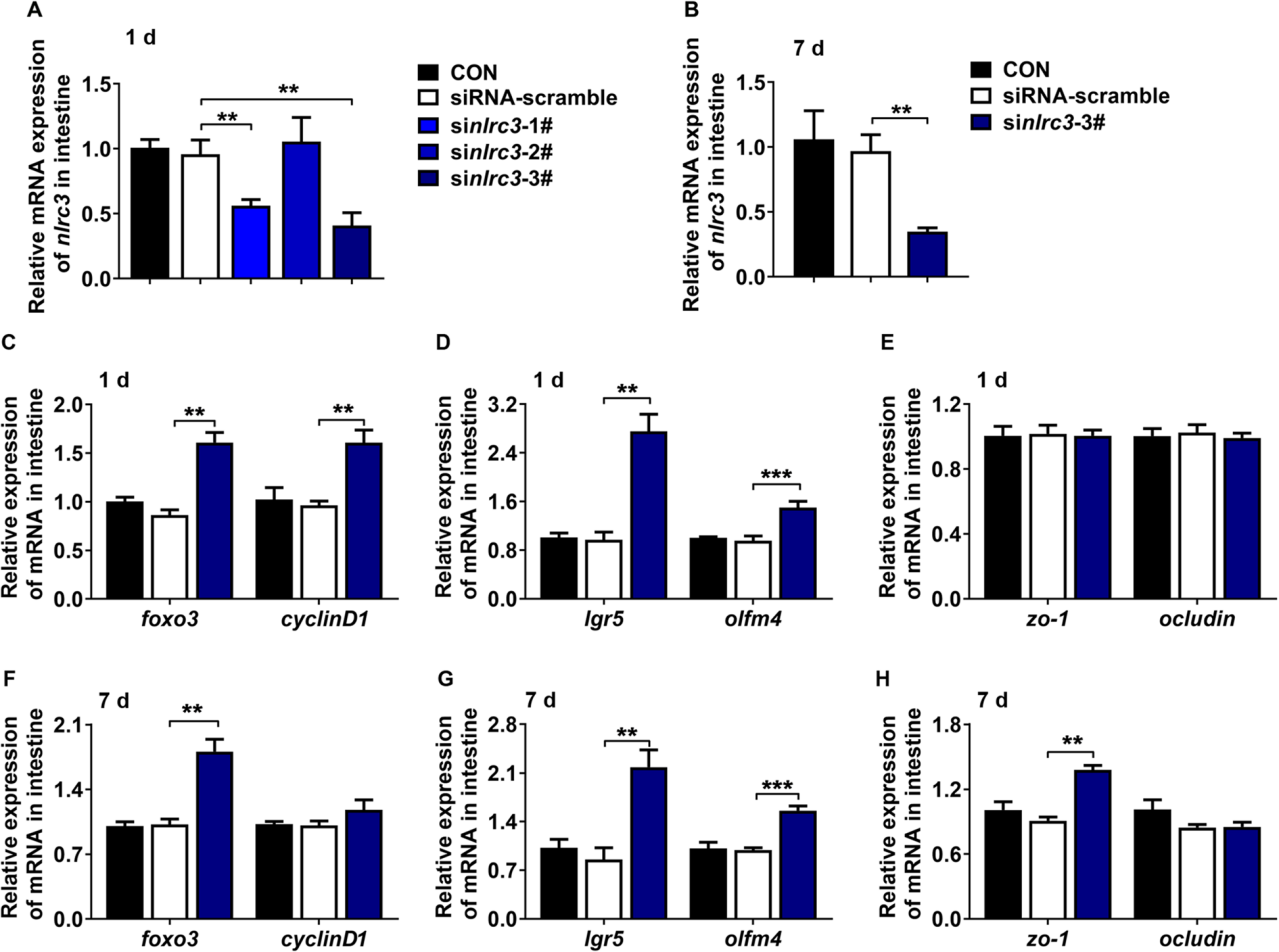
Nlrc3 was the key factor to protect gut barrier integrity. **A** In vivo si*nlrc3* interference for 1 d; **B** In vivo si*nlrc3* interference for 7 d; **C** and **F** The relative gene expression of *foxo3* and *cylinD1* in the short-term and long-term experiments; **D** and **G** The relative gene expression of *lgr5* and *olfm4* in the short-term and long-term experiments; **E** and **H** The relative gene expression of *zo-1* and *ocludin* in the short-term and long-term experiments (*n* = 5). Data are represented as mean ± SEM. Significant difference compared with siRNA-scramble group (Student’s* t*-test; ^**^P < 0.01, ^***^P < 0.001). CON, control; *nlrc3*, NLR family CARD domain containing 3; *foxo3*, forkhead box O3; *lgr5*, leucine-rich repeat-containing G protein-coupled receptor 5; *olfm4*, olfactomedin 4; *zo-1*, zona occludens 1

We designed a short-term experiment (si*nlrc3*–3# interference for 1 d) and a long-term experiment (si*nlrc3*–3# interference for 7 d) in vivo to detect how the inhibition of *nlrc3* modulate the gene expression of ISC markers. si*nlrc3*–3# had the best inhibition effect of intestinal *nlrc3* (*P* < 0.05, Fig. 4A), which was further used in both short-term and long-term experiments. The gene expression of *nlrc3* was also significantly inhibited by si*nlrc3*–3# in the long-term experiment (*P* < 0.05, Fig. 4B). The expression levels of *foxo3*, *cyclinD1* were upregulated when *nlrc3* was knock down (*P* < 0.05, Fig. 4C and F), suggesting that the expression of *foxo3* and *cyclinD1* could be influenced by *nlrc3*. The gene expression of ISC markers (*lgr5* and *olfm4*) were significantly promoted after si*nlrc3* interference (*P* < 0.05, Fig. 4D and G). There was no difference in the expression levels of *zo-1* and *ocludin* in the short-term experiment (*P* > 0.05, Fig. 4E). However, the expression level of *zo-1* was significantly higher in the long-term experiment (*P* < 0.05, Fig. 4H). These results indicated that the inhibition of *nlrc3* had a time-delay effect on promoting the expression of *zo-1* compared with the effect on ISC markers expression.

### Addition of *P. pentosaceus* YC altered the composition of gut microbiota

**Fig. 5:**
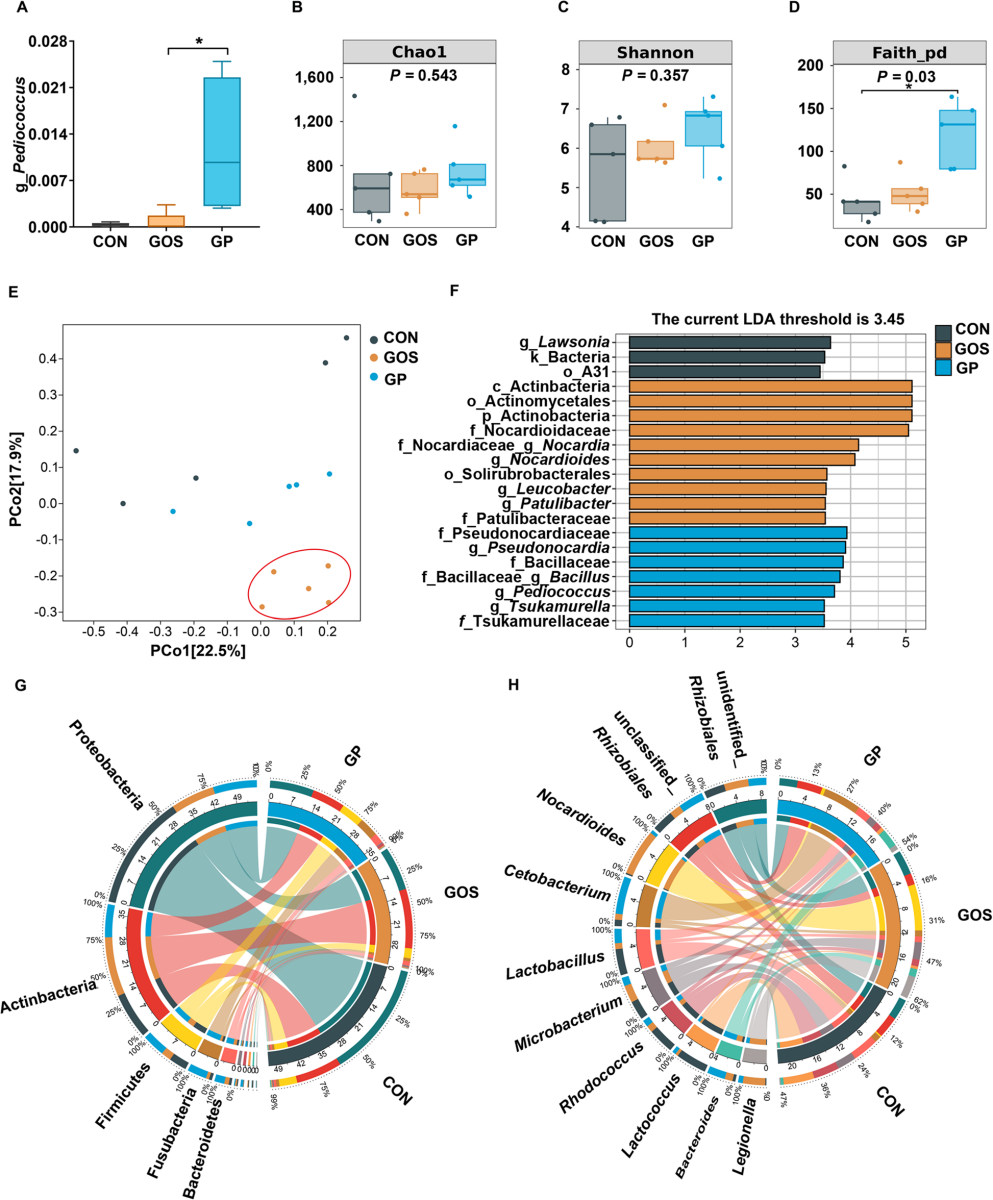
Addition of *P. pentosaceus* YC altered the composition of gut microbiota. **A** The relative abandance of *Pediococcus*; **B** Chao1 index. **C** Shannon index; **D** Faith_pd index; **E** PCoA analysis; **F** LEfSe analysis; **G** Circos analysis in the level of phylum; **H** Circos analysis in the level of genus (*n* = 5). Data are represented as mean ± SEM. Significant difference of the relative abandance of *Pediococcus* compared with GOS group (ANOVA with Tukey’s test; ^*^P < 0.05) and significant difference of Faith_pd (Kruskal-Wallis test; ^*^P < 0.05). CON, control diet; GOS, gossypol diet; GP, gossypol diet supplemented with *P. pentosaceus* YC; PCoA, principal coordinate analysis; LDA, laser diffraction analysis

16S rRNA sequencing was conducted to detect whether addition of *P. pentosaceus* YC altered gut microbiota composition. The abundance of *Pediococcus* was significantly increased in GP group, indicating the successful colonization of *P. pentosaceus* YC in fish gut (*P* < 0.05, Fig. 5A). Dietary *P. pentosaceus* YC had no significant effects on Chao1 (characterized abundance) and Shannon (characterized diversity) indexes (*P* > 0.05, Fig. 5B and C), but significantly increased Faith_pd (characterized phylogenetic diversity) index (*P* < 0.05, Fig. 5D). Addition of *P. pentosaceus* YC recovered the composition of gut microbiota similar to that of the CON group (PCo1 and PCo2 were 22.5% and 17.9%, Fig. 5E). LEfSe analysis indicated that Actinobacteria and Nocardioidaceae were dominant in GOS group, and Pseudonocardiaceae, Bacillaceae and *Pediococcus* were predominant in GP group (Fig. 5F). Proteobacteria, Actinobacteria, Firmicutes, Fusobacteria, and Bacteroidetes were the major phyla across all groups, and addition of *P. pentosaceus* YC increased the abundance of Firmicutes, Fusobacteria, Bacteroidetes, but decreased the abundance of Actinobacteria at the phylum level (Fig. 5G). *Rhizobiales*, *Nocardioides*, *Cetobacterium*, and *Lactobacillus* were the dominant genera in all three groups, and addition of *P. pentosaceus* YC increased the abundance of *Lactobacillus*, *Cetobacterium*, *Bacteroides*, and decreased the abundance of *Nocardioides* and *Legionella* at the genus level (Fig. 5H). Collectively, addition of *P. pentosaceus* YC altered the intestinal microbial composition.

### Addition of *P. pentosaceus* YC increased the propionate content in gut

**Fig. 6:**
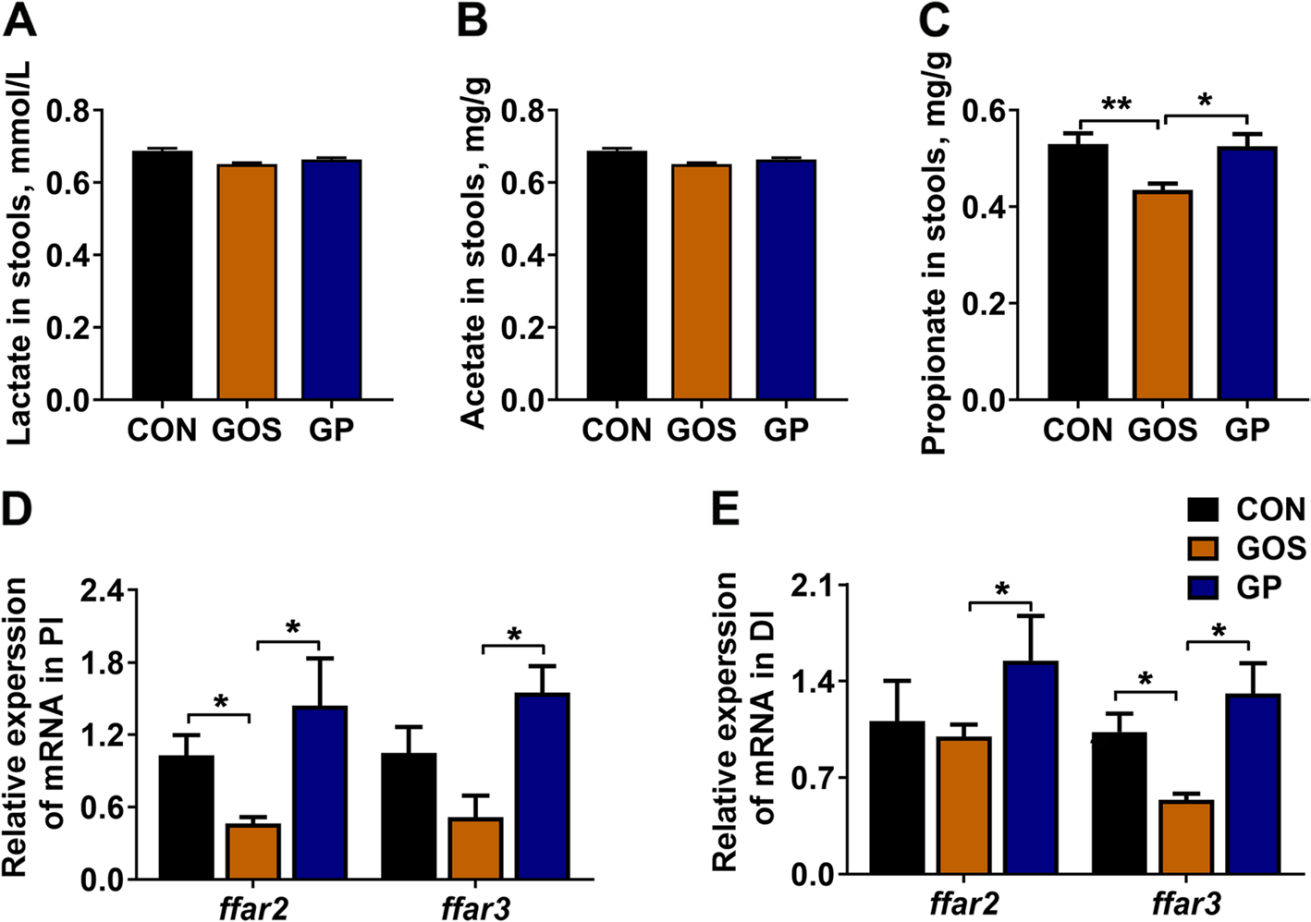
Addition of *P. pentosaceus* YC induced the accumulation of gut propionate. **A** The levels of lactate in the intestinal contents; **B** The levels of acetate in the intestinal contents; **C** The levels of propionate in the intestinal contents; **D** and **E** The relative genes expression of *ffar2* and *ffar3* in PI and DI (*n* = 6). Data are represented as mean ± SEM. Asterisk refers to the significant difference (ANOVA with Tukey’s test; ^*^P < 0.05, ^**^P < 0.01). CON, control diet; GOS, gossypol diet; GP, gossypol diet supplemented with *P. pentosaceus* YC; *ffar*, free fatty acid receptor; PI, proximal intestine; DI, distal intestine

Considering that addition of *P. pentosaceus* YC altered the intestinal microbiota composition, we detected the content of microbial derived acetate, propionate, butyrate, and lactate in the intestinal contents. Butyrate was too low to be detected and there were no notably changes in the levels of lactate and acetate among three groups (*P* > 0.05, Fig. 6A and B). A significant decrease of propionate was observed in GOS group, and addition of *P. pentosaceus* YC restored the level of propionate in GP group (*P* < 0.05, Fig. 6C). Moreover, the expression levels of free fatty acids receptor 2 (*ffar2*, also known as G protein-coupled receptor (*GPR*)*43*) and *ffar3* (also known as *GPR41*) increased remarkably in the PI and DI after *P. pentosaceus* YC treatment (*P* < 0.05, Fig. 6D and E). In conclusion, addition of *P. pentosaceus* YC increased intestinal propionate content and upregulated the expression of *ffar2* and *ffar3*.

### Propionate stimulated wound healing in vitro

**Fig. 7:**
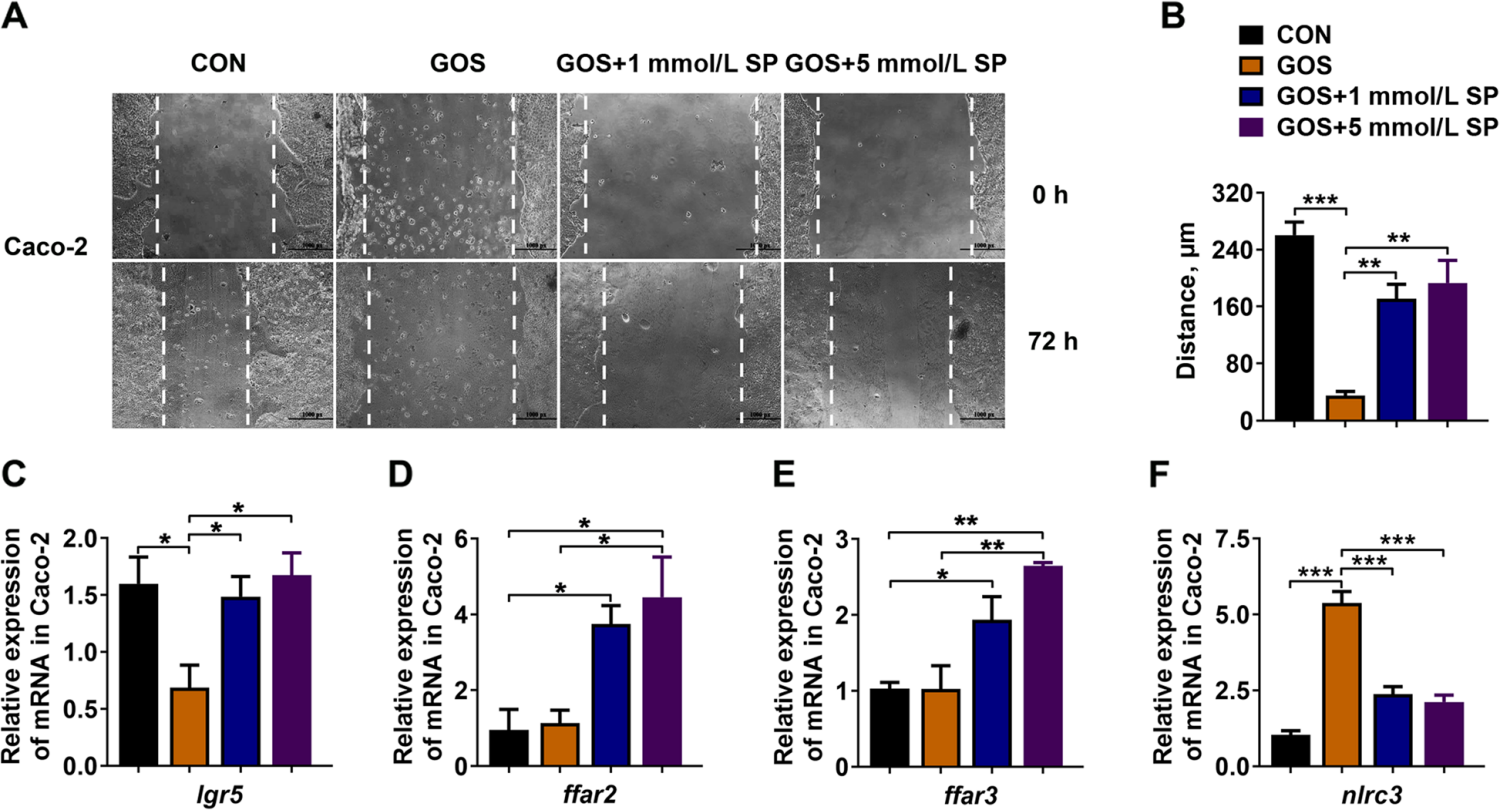
Propionate improved the wound healing of Caco-2 cells in vitro. **A** Representative phase contrast images of Caco-2 cells, scale bar = 1,000 px (1 px = 4.23 μm); **B** Quantification of average migration distance; **C–F** The relative gene expression of *lgr5*, *ffar2*, *ffar3* and *nlrc3* in Caco-2 cells (*n* = 3). Asterisk refers to the significant difference (ANOVA with Tukey’s test; ^*^P < 0.05, ^**^P < 0.01, ^***^P < 0.001). CON, control; GOS, gossypol; SP, sodium propionate; *lgr5*, leucine-rich repeat-containing G protein-coupled receptor 5; *ffar*, free fatty acid receptor; *nlrc3*, NLR family CARD domain containing 3

The wound-healing assay of Caco-2 cells was conducted to detect the reparative function of propionate. Results revealed that gossypol suppressed the wound healing, but supplementation of sodium propionate strikingly accelerated the healing of wound (*P* < 0.05, Fig. 7A and B). We further detected the above mentioned differentially expressed genes on the Caco-2 cells. Here, we found that *lgr5* had a significantly higher expression after addition of sodium propionate compared to the GOS group, which indicated that propionate might enhance stem cell proliferation during the healing process (*P* < 0.05, Fig. 7C). Furthermore, we examined the expression of *ffar2* and *ffar3*, and found these two genes were significantly upregulated in sodium propionate treatment (*P* < 0.05, Fig. 7D and E). The expression of *nlrc3* was dramatically increased in GOS group, and notably decreased by sodium propionate administration (*P* < 0.05, Fig. 7F). These results indicated that propionate inhibited *nlrc3* expression, promoted *lgr5* expression, and improved the wound healing of Caco-2 cells.

## Discussion

Dietary components and xenobiotics have the adverse effects on the intestinal health [46–48]. Gossypol, a toxic compound contained in CSM, has detrimental effects on the growth condition, intestinal immunity and barrier barrier integrity in various animals, how to decrease the deleterious effects of gossypol residue remains to be expolored [13, 17, 19–22]. Probiotics have the potential to promote the growth condition and repair the gut barrier, and concomitantly, intestinal microbiota is involved [34, 36, 49–51]. The present research revealed that the weight gain, specific growth rate and final body length were significantly augmented after the addition of *P. pentosaceus* YC, suggesting its ability to restore the growth retardation caused by gossypol in Nile tilapia. The increased growth may be attributed to the repair of gut barrier, as intestinal structure integrity is indispensable for the absorption of nutrients and resisting of pathogens [52]. Wang et al. [17] reported that dietary gossypol resulted in the loose arrangement of enterocyte and epithelial sloughing to damage the structure of intestinal epithelial cells in grass carp (*Ctenopharyngodon idella*), but there is no effective stretagy to reduce the negative effect of gossypol. Our previous study in Nile tilapia proved that the disruption of intestinal microbial homeostasis was the reason for gossypol to exacerbate gut barrier injury [21]. And in the present study, we found that addtion of *P. pentosaceus* could improve the intesitnal strucuture and integrity when fish were exposed to gossypol.

ISC proliferation is fundamental to sustaining gut barrier integrity by governing the rapid renewal of intestinal epithelium, particularly in response to gut barrier injury [32, 34, 53]. Several signaling pathways including NLR, Wnt, bone morphogenetic protein (BMP) and Notch signals have been documented to regulate ISC proliferation [32]. Here, the RNAseq analysis inspired us to focus on the Nlrc3 (also named CLR16.2 and Nod3), a molecular belonging to NLRs family [54]. NOD1 and NOD2 in the NLRs family are capable of regulating ISC proliferation [55–57]. However, the link between Nlrc3 and ISC proliferation has not been explored. Research has discovered that a deficiency of NLRC3 spurred cell proliferation in wound and hastened the healing of murine cutaneous wound [58]. Correspondingly, the results of the feeding trail substantiated that the expression of Nlrc3 had a negative correlation with the expression of ISC markers, *lgr5* and *olfm4*, and the siRNA interference experiment indicated that inhibition of Nlrc3 could stimulate the expression of these genes, potentially implying ISC proliferation. *Foxo3* and *cyclinD1* were two downstream genes of *nlrc3* [59], and *cyclinD1* was found to contribute to porcine ISC proliferation [35]. In this study, the expression levels of *foxo3* and *cyclinD1* were up-regulated when *nlrc3* was inhibited, suggesting that the effect of *nlrc3* on *foxo3* and *cyclinD1* was conserved in different animals. The proliferated ISCs could move up and divide into epithelial cells to replace the damaged cells in the villi [60]. We also found the expression of *si* (a marker of terminal differentiation in enterocytes) and *villin* (enterocytes marker) increased when *P. pentosaceus* YC was added (Fig. S1), suggesting that the addition of *P. pentosaceus* YC may also influence the differentiation to enterocytes, yet this requires more validation. Furthermore, it has been revealed that mice lacking NLRC3 were predisposed to cancer due to hyperproliferation [59], so we identified whether addition of *P. pentosaceus* YC with standard diet caused harmful effect to the host. The results showed that addition of *P. pentosaceus* YC did not influence the growth performance or intestinal barrier function (Fig. S2), indicating *P. pentosaceus* YC did not induce the excessive proliferation under the normal physiological condition of fish.

The intestinal microbiota and the derived metabolites can initiate signals of hosts to maintain the gut barrier integrity [61, 62]. Previous study has demonstrated that addition of *P. pentosaceus* altered the gut microbiota composition in mice and zebrafish [29–31, 38]. Consistently, *P. pentosaceus* YC administration raised the abundance of Firmicutes, Fusobacteria, Bacteroidetes phyla and *Lactobacillus*, *Cetobacterium*, and *Bacteroides* genera. These bacteria are capable of effectively producing SCFAs, which can help to repair intestinal barrier damage [63–65]. The metabolites analysis further indicated that the addition of *P. pentosaceus* YC increased the level of propionate significantly. Unlike the beneficial effect of acetate and butyrate in maintaining intestinal barrier [66, 67], the effect of propionate on gut barrier was inconsistent [68, 69]. It has been found that propionate supplementation in high-fat diet induced intestine damage in zebrafish [68], but sodium propionate supplementation in the diet with high soybean meal promoted the growth performance of turbot and enhanced the expression of intestinal tight junction proteins [69]. In our study, exogenous addition of sodium propionate promoted wound healing of Caco-2 cells, insinuating the reparative effect on gut barrier integrity of propionate with the presence of gossypol.

Caco-2 cells line was used because it has a classical intestinal crypt stem cell-like population and can differentiate into intestinal epithelial-like cells, which is an ideal model to research the connection between ISCs and intestinal epithelium [70, 71]. With the addition of sodium propionate, we discovered a high expression of LGR5 [70], a marker of the classic intestinal crypt stem cell-like population of Caco-2 cells, indicating stem cell proliferation. GPR41 and GPR43 are known to mediate propionate to promote murine intestinal stem cell proliferation [72]. Moreover, SCFAs has been proved to inhibit NLRs by activating GPRs [73]. Here, propionate increased *ffar2*/*ffar3* expression and decreased *nlrc3* expression in vitro and in vivo, indicating propionate may inhibit Nlrc3 through GPRs. Taken together, addition of *P. pentosaceus* YC increased the level of gut microbiota-derived propionate and repaired gossypol-induced intestinal barrier injury in Nile tilapia.

## Conclusions

In conclusion, the present study established that *P. pentosaceus* YC had the protective effect on repairing gossypol-induced intestinal barrier injury. Addition of *P. pentosaceus* YC altered the gut microbiota composition and increased intestinal propionate to inhibit Nlrc3, up-regulated the genes of ISC proliferation markers and repaired intestinal barrier injury. This study provides a potential strategy for gossypol-induced gut barrier injury, which will also benefit the application of CSM in the future.

### Supplementary Information

**Additional file 1: Table S1.** Ingredient formulation and proximate composition of the experimental diets (dry matter basis). **Table S2.** Primer pair sequences and product size of the genes used for qPCR. **Table S3.** *nlrc3* siRNA information. **Fig. S1.** Addition of *P. pentosaceus* YC promoted the gene expression of enterocytes. **Fig. S2.** Administration of *P. pentosaceus* YC have no effect on Nile tilapia fed with the control diet. **Supplementary methods.** Animal experiments, proximate composition analysis, high liquid chromatography analysis of gossypol.

## Data Availability

All data supporting our findings are included in the manuscript.

## Abbreviations

ANOVA: One-way analysis of variance
BCA: Bicinchoninic acid
BMP: Bone morphogenetic protein
CARD: Caspase recruitment domain
CF: Condition factor
CFU: Colony-forming unit
CON: Control diet
CSM: Cottonseed meal
DEG: Differentially expressed gene
DI: Distal intestine
ef1α: Elongation factor 1 alpha
ELISA: Enzyme-linked immunosorbent assays
FBW: Final body weight
FC: Fold change
ffar: Free fatty acid receptor
FM: Fish meal
foxo3: Forkhead box O3
GC-MS: Gas chromatography-mass spectrometry
GOS: Gossypol diet
GP: Gossypol diet supplemented with *P. pentosaceus* YC
H&E: Hematoxylin and eosin
HSI: Hepatosomatic index
IBW: Initial body weight
il-1β: Interleukin-1β
ISC: Intestinal stem cell
iκbκb: Inhibitor of nuclear factor kappa B kinase
KEGG: Kyoto Encyclopedia of Genes and Genomes
LDA: Laser diffraction analysis
LEfSe: Linear discriminant analysis effect size
Lgr5: Leucine-rich repeat-containing G-protein coupled receptor 5
LPS: Lipopolysaccharide
MFI: Mesenteric fat index
MRS: Man Rogosa Sharpe
Nlrc3: NLR family CARD domain containing 3
NLR: NOD-like receptors
Olfm4: Olfactomedin 4
PBS: Phosphate buffered saline
PCoA: Principal coordinate analysis
PI: Proximal intestine
PMSF: Phenylmethylsulfonyl fluoride
qPCR: Quantitative PCR
RIPA: Radioimmunoprecipitation assay
SBM: Soybean meal
SCFA: Short chain fatty acid
SDS: Sodium dodecyl sulfate
SDS-PAGE: Sodium dodecyl sulfate polyacrylamide gel electrophoresis
SEM: Standard error of mean
SGR: Specific growth rate
SP: Sodium propionate
TEER: Transepithelial electrical resistance
WG: Weight gain
zo-1: Zona occludens 1
