# Supplementary material for: Probiotic Pediococcus pentosaceus restored gossypol-induced intestinal barrier injury by increasing propionate content in Nile tilapia
Source: J Anim Sci Biotechnol. 2024 Apr 7;15:54. doi: 10.1186/s40104-024-01011-w (PMC10999087; doi:10.1186/s40104-024-01011-w)
Supplement: Supplementary file 1 — Additional file 1: Table S1. Ingredient formulation and proximate composition of the experimental diets (dry matter basis). Table S2. Primer pair sequences and product size of the genes used for qPCR. Table S3. nlrc3 siRNA information. Fig. S1. Addition of P. pentosaceus YC promoted the gene expression of enterocytes. Fig. S2. Administration of P. pentosaceus YC have no effect on Nile tilapia fed with the control diet. Supplementary methods. Animal experiments, proximate composition analysis, high liquid chromatography analysis of gossypol. [file 40104_2024_1011_MOESM1_ESM.docx]

**Additional file 1**

**Table S1** Ingredient formulation and proximate composition of the experimental diets (dry matter basis)

| **Item** | **CON** | **GOS** | **GP** | **CP** |
| --- | --- | --- | --- | --- |
| Ingredients | | | | |
| Casein, g/kg | 344 | 344 | 344 | 344 |
| Gelatin, g/kg | 86 | 86 | 86 | 86 |
| Soyabean oil, g/kg | 60 | 60 | 60 | 60 |
| Maize starch, g/kg | 300 | 300 | 300 | 300 |
| Vitamin premix^*^, g/kg | 12 | 12 | 12 | 12 |
| Mineral premix^†^, g/kg | 12 | 12 | 12 | 12 |
| Ca(H_2_PO_4_)_2_, g/kg | 10 | 10 | 10 | 10 |
| Carboxymethylcellulose, g/kg | 30 | 30 | 30 | 30 |
| Cellulose, g/kg | 139.75 | 139.45 | 139.45 | 139.75 |
| Choline chloride, g/kg | 5 | 5 | 5 | 5 |
| Dimethyl-β-propiothetin, g/kg | 1 | 1 | 1 | 1 |
| Butylated hydroxytoluene, g/kg | 0.25 | 0.25 | 0.25 | 0.25 |
| Gossypol, g/kg | 0 | 0.3 | 0.3 | 0 |
| *P. pentosaceustosaceus*, CFU/g | 0 | 0 | 1×10^8^ | 1×10^8^ |
| Total quantity, g | 1000 | 1000 | 1000 | 1000 |
| Proximate composition, % | | | |  |
| Moisture | 10.37±0.35 | 9.65±0.22 | 10.14±0.25 | 10.57±0.19 |
| Crude protein | 37.78±0.51 | 37.56±0.69 | 37.43±0.54 | 37.63±0.15 |
| Crude lipid | 6.10±0.02 | 5.93±0.41 | 5.89±0.14 | 5.92±0.10 |
| Crude ash | 3.19±0.14 | 2.96±0.01 | 2.99±0.05 | 2.95±0.05 |
| Gossypol, mg/kg | 0 | 288±0.26 | 279±0.34 | 0 |

*CON* Control diet, *GOS* Gossypol diet, *GP* Gossypol diet supplemented with *P. pentosaceus* YC, *CP* Control diet supplemented with *P. pentosaceus* YC

^*^ Vitamin premix (Hangzhou Minsheng Bio-Tech Co., Ltd., Hangzhou, China) (mg or IU/kg vitamin premix): 500,000 IU vitamin A, 50,000 IU vitamin D_3_, 2,500 mg vitamin E, 1,000 mg vitamin K_3_, 5,000 mg vitamin B_1_, 5,000 mg vitamin B_2_, 5,000 mg vitamin B_6_, 5,000 mg vitamin B_12_, 25,000 mg inositol, 10,000 mg pantothenic acid, 100,000 mg choline, 25,000 mg niacin, 1,000 mg folic acid, 250 mg biotin, 10,000 mg vitamin C

^†^ Mineral premix (Hangzhou Minsheng Bio-Tech Co., Ltd., Hangzhou, China) (g/kg mineral premix): 147.4 g MgSO_4_·7H_2_O; 49.8 g NaCl; 10.9 g Fe (II) gluconate; 3.12 g MnSO_4_·H_2_O; ZnSO_4_·7H_2_O; 0.62 g CuSO_4_·5H_2_O; 0.16 g KI; 0.08 g CoCl_2_·6H_2_O; 0.06 g NH_4_ molybdate; 0.02 g NaSeO_3_

**Table S2** Primer pair sequences of the genes used for qPCR

| **Gene** | **Primer sequence (5´→3´)** | **GenBank No.** |
| --- | --- | --- |
| *ef1α* | ATCAAGAAGATCGGCTACAACCCT  ATCCCTTGAACCAGCTCATCTTGT | KJ123689 |
| *β-actin* | AGCCTTCCTTCCTTGGTATGGAAT  TGTTGGCGTACAGGTCCTTACG | KJ126772 |
| *occludin* | GTGTTGCTGCTTTCTTCGCT  GTGTTGCTGCTTTCTTCGCT | XM_003445131 |
| *Zo-1* | CCGCAGATCAGTCCCTCTTC  GTACGGAGTTAGCATCGCCA | XM_013270540 |
| *claudin* | GAGGAGTCAGTCGGAGTCT  CAGCACCGTCTTGAACTTG | XM_003448981 |
| *cadherin1* | CAGGGCATGTCAAAGACCAG  TACGGCCTAAGCATGTCGG | XM_019366361 |
| *si* | ACTACAACTCCCCCTCCTCC  CCCACCCTTCACATACACAGTC | XM_025902524 |
| *villin* | TGGATTAGGCATTACCGAGCC  AGTCCTGGCTGGTTCCTTTG | XM_005448864 |
| *nlrc3* | GACTGTACCAGGACAAGGTGT  GGACTTTGGACACCCCTGCT | XM_025903452 |
| *foxo3* | CGGTCGCCTCTCACCTATTC  AGCCTTCCCATTCGTCTTGG | XM_005454618 |
| *cyclinD1* | CCAATGGGGTGCGTCTTACT  GCTCTCCTTGCTCGTGGTAA | XM_003459284 |
| *lgr5* | AGCAGAGACACACCGCCTAT  CGGATGGAAAGTGGAGCACA | XM_040036044 |
| *olfm4* | CAGATACGCCCACTACGTCC  GGACCTTGGATGGTGTTGGT | XM_003439551 |
| *ffar2* | TGCCCGACAGATCAAACCTC  TCAATCGGGGTGGGTTTCTG | XM_013274562 |
| *ffar3* | CCTCCTCTCTTTGTTAGTGCC  ATATCATCTCCCAGCAACCGC | XM_019350661 |
| *Hsef1α* | TCCTACCACCAACTCGTCCA | NM_001402 |
|  | TTGCCACGACGAACATCCTT |  |
| *Hsβ-actin* | ATTGGCAATGAGCGGTTCC  GGTAGTTTCGTGGATGCCACA | NM_001101 |
| *Hslgr5* | AGGTCTGGTGTGTTGCTGAG  GTGAAGACGCTGAGGTTGGA | AF061444 |
| *Hsffar2* | CTGGACCCCCTGCTCTTCTA  CCCTGTCCTCATTTGTCCCC | AB378083 |
| *Hsffar3* | TGCACTAGGTCTGGAGAGACA  CGGAGAAGTAGGACTGGTCG | NM_005304 |
| *Hsnlrc3* | CAGGAGCCTCACCAGCTTAG  TGAGGCCACCTGGAGATAGA | FJ889357 |

*Ef1α* Elongation factor 1 alpha, *zo-1* Zona occludens 1, *si* Sucrase-isomaltase, *nlrc3* NLR family CARD domain containing 3, *foxo3* Forkhead box O3, *lgr5* Leucine-rich repeat-containing G protein-coupled receptor 5, *olfm4* Olfactomedin 4, *ffar* Free fatty acid receptor, Hs Homo sapiens

**Table S3** *nlrc3* siRNA information

|  | **Sequence ((5´→3´)** |
| --- | --- |
| Scramble siRNA | sense: UUCUCCGAACGUGUCACGUTT  anti-sense: CGUGACACGUUCGGAGAATT |
| Target site1 | sense: GGGCUUCUUAAACACAAGUTT  anti-sense: CUUGUGUUUAAGAAGCCCTT |
| Target site2 | sense:GUGCCAAAGACUGCCAUAUTT  anti-sense: UAUGGCAGUCUUUGGCACTT |
| Target site3 | sense: GCUGGUGCAAUCAAGUUUATT  anti-sense: AAACUUGAUUGCACCAGCTT |

**
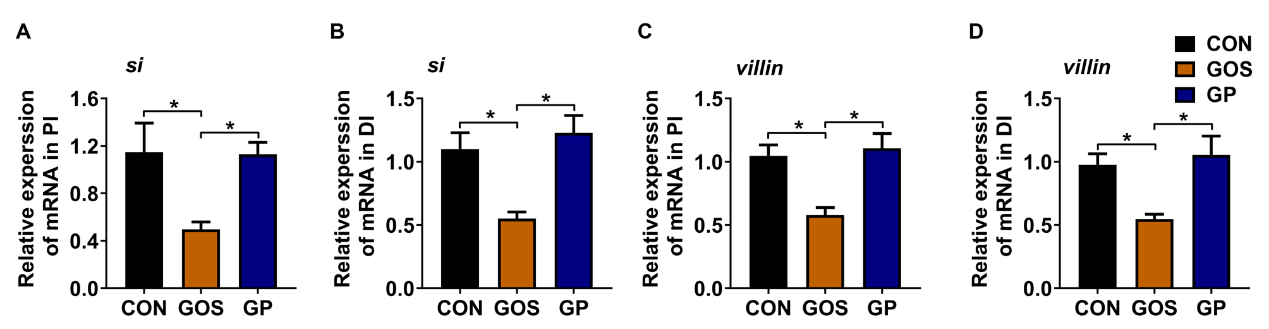
**

**Fig. S1** Addition of *P. pentosaceus* YC promoted the genes expression of enterocytes. **A** and **B** The gene expression of *si* in PI and DI; **C** and **D** The gene expression of *villin* in PI and DI (*n* = 6). Data are represented as mean ± SEM. Asterisk refers to the significant difference (ANOVA with Tukey’s test; ^*^*P* < 0.05). CON, control diet; GOS, gossypol diet; GP, gossypol diet supplemented with *P. pentosaceus* YC; *si*, sucrase-isomaltase; PI, proximal intestine, DI, distal intestine

**
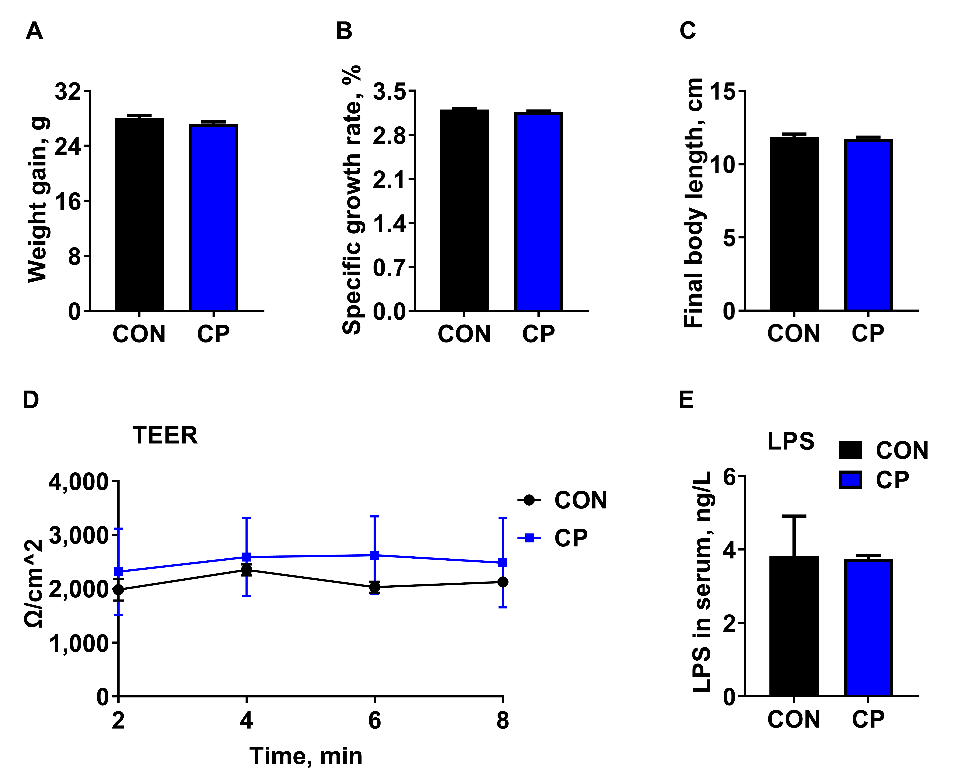
**

**Fig. S2** Administration of *P. pentosaceus* YC have no effect on Nile tilapia fed with the control diet. **A** Weight gain, **B** Specific growth rate, **C** Final body length, **D** TEER of intestine, **E** LPS concentrations in serum. Data are represented as mean ± SEM. Significant difference were analyzed by Student’s *t*-test. CON, control, GP, control diet supplemented with *P. pentosaceus* YC, TEER, transepithelial electrical resistance, LPS, lipopolysaccharide

**Supplementary methods**

**Animal experiments**

Fish in the CP group were fed pellets mixed CON diet with 1.0×10^8^ CFU/g *P. pentosaceus* YC for 10 weeks. The formulation of the diet was listed in Table S1.

**Proximate composition analysis**

The moisture, crude protein, crude lipid and crude ash contents of diets were determined by standard methods as described by AOAC (2016). Moisture content was measured by oven-drying at 105 °C until attained to constant weight. Crude protein and crude lipid were separately determined by the Kjeldahl method using Kjeldahl apparatus and manual titration and chloroform/methanol method. Crude ash contents were estimated by incinerating in a muffle furnace at 550 °C for 6 h. The proximate composition of diets was provided individually in Table S1.

**High liquid chromatography (HPLC) analysis of gossypol**

The gossypol contents in diets were detected by HPLC. Briefly, diets were homogenized with acetone for 1 min before crushed with ultrasonic wave at 40 °C for 30 min. After centrifuging at 3,000 × *g* for 10 min at 4 °C, the supernatants were dissolved with mobile phase (chromatographic acetonitrile/0.2% phosphoric acid, 85/15, v/v) and filtered using a syringe filter (0.45 μm). The HPLC analysis was performed on an LC-20A Liquid Chromatograph-C18 column (Shimadzu, Kyoto, Japan) and detected at 235 nm for 10 min at 25 °C with a flowrate of 1.0 mL/min. The gossypol levels were calculated to gossypol-acetic acid (G4382, Sigma-Aldrich, St. Louis MO, USA) standard line and showed in the Table S1.
